# Antibiotic Coordination Frameworks against Antibiotic Resistance: How to Involve Students through Experimental Practices in the Search for Solutions to Public Health Problems

**DOI:** 10.1021/acs.jchemed.3c01125

**Published:** 2024-03-06

**Authors:** Eva María Domínguez-Martín, Epole Ntungwe, Vera M. S. Isca, Salvatore Princiotto, Ana María Díaz-Lanza, Vânia André, Patrícia Ríjo

**Affiliations:** ¶ CBIOS − Universidade Lusófona’s Research Center for Biosciences & Health Technologies, Campo Grande 376, 1749-024 Lisbon, Portugal; ‡ Universidad de Alcalá de Henares, Facultad de Farmacia, Departamento de Ciencias Biomédicas (Área de Farmacología); Nuevos Agentes Antitumorales, Acción Tóxica Sobre Células Leucémicas, Ctra. Madrid-Barcelona km. 33,600, 28805 Alcalá de Henares, Madrid, España; § Instituto de Investigação do Medicamento (iMed.ULisboa), Faculdade de Farmácia, Universidade de Lisboa, 1649-003 Lisbon, Portugal; ∥ Department of Food, Environmental and Nutritional Sciences (DeFENS), University of Milan, Via Celoria 2, Milan 20133, Italy; ⊥ Centro de Química Estrutural, Institute of Molecular Sciences, Instituto Superior Técnico, Universidade de Lisboa, Avenida Rovisco Pais, 1049-001 Lisbon, Portugal

**Keywords:** Laboratory Experiment, Second Year/Upper-Division Undergraduate, Inorganic Chemistry, Hands-On Learning, Antibiotic Coordination Framework (ACF), Medicinal Chemistry

## Abstract

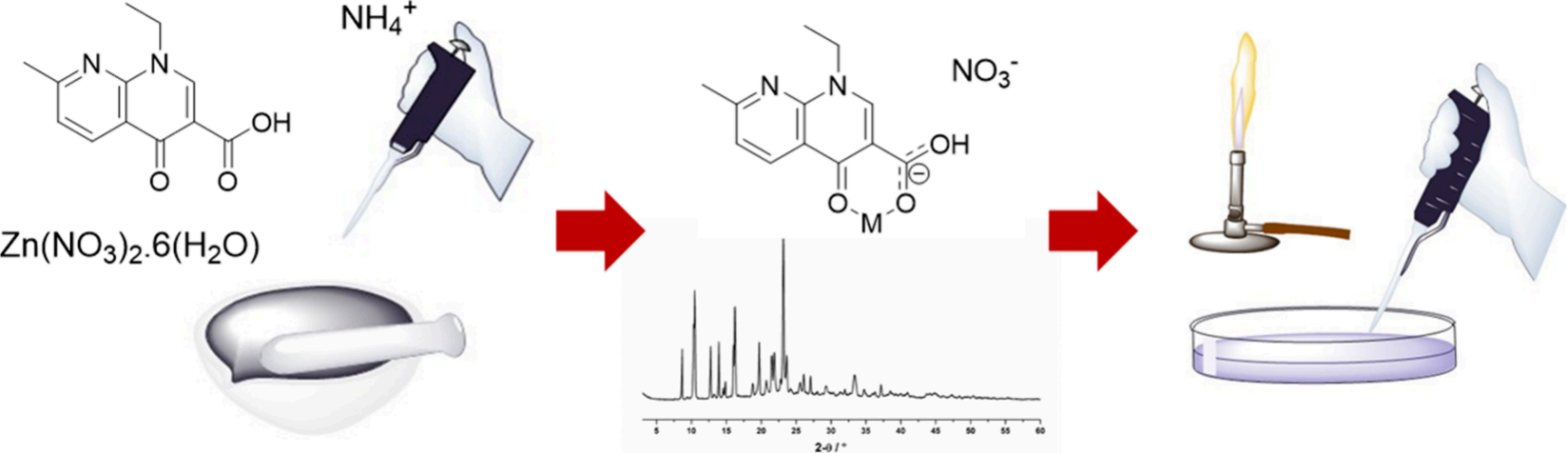

For decades, multiple varieties of antibiotics have been successfully used for therapeutic purposes. Nevertheless, antibiotic resistance is currently one of the major threats to global health. This work presents an innovative laboratory practice carried out in an inorganic medicinal chemistry course within the Degrees of Pharmacy and Biochemistry for undergraduate students. This experiment includes three classes of 2 h each. The first class consisted of the mechanochemical synthesis of an antibiotic coordination framework (ACF) using a known antibiotic (nalidixic acid) and zinc as the ligand. The prepared Zn-nalidixic acid ACF (Zn-ACF) was obtained in up to 82% yield with high purity. On the second day, the synthesized Zn-ACF was characterized by Fourier-transform infrared spectroscopy (FTIR) and powder X-ray diffraction (PXRD). Finally, during the last class, the antimicrobial activity was tested against *Escherichia coli* by the well diffusion method. The students verified the higher antimicrobial activity of Zn-ACF compared to nalidixic acid, proving that small changes in the chemical structure can result in great biological differences. In the end, the students presented their results in a poster format, encouraging the development of their soft skills and scientific results communication and dissemination. In the future, it is expected that such a laboratory experiment at the interface between medicinal chemistry, microbiology, analytical techniques, public health, and pharmacology will lead to the development and implementation of some service-learning practices and will serve as a model to look at for other courses and institutions.

## Introduction

The inclusion of effective antibacterial therapies for the treatment of infectious diseases has completely changed clinical practices and significantly assisted the advent of modern medicine as we know it.^1^

**Figure 1:**
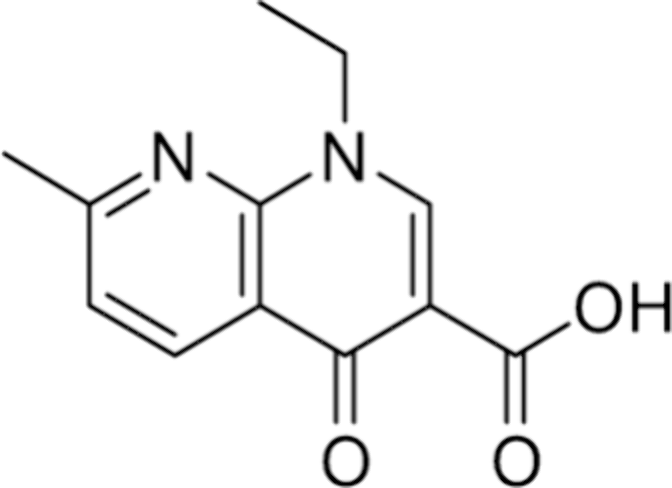
Chemical structure of nalidixic acid.

Although it was only in the period spanning the 1940s to the 1960s that many antibiotics were discovered and used, beginning the golden age of the antibiotics,^2^ nalidixic acid (Figure 1) was described for the first time in 1962, when Lesher et al. reported it as a byproduct in the synthesis of antimalarial quinine derivatives.^3−5^ Nalidixic acid was the first quinolone antibiotic ever discovered and was approved in 1967 for the treatment of uncomplicated urinary tract infections (UTIs), such as those caused by Gram-negative *Escherichia coli* (*E. coli*) and *Klebsiella*.^6^ It acts as a bacteriostatic agent at low concentrations and as a bactericidal agent at higher doses, mainly because of its activity as a DNA gyrase inhibitor. As a consequence, the formation of a relaxation complex between the enzyme and bacterial DNA is favored, preventing the replication of the Gram-negative bacteria.^6,7^ Despite these properties, its use is now limited due to the narrow spectrum of activity, low serum concentrations achieved, high inhibitory concentrations required, and several adverse effects.^6^

Currently, due to overuse and misuse of many antibiotics, pathogenic bacteria acquired significant resistance against antibiotics, and the class of quinolones was no exception. In terms of mechanisms, quinolone resistance is mediated by one or a combination of mutations in target-site genes and consequent lack of drug-binding affinity toward target enzymes.^8^ An alternative pathway for surmounting this challenge is modifying the currently available antibiotics, aiming to introduce new properties or enhance the activity of current drugs. Coordination complexes with pharmacologically active molecules, including metallodrugs, metallopharmaceuticals, and antibiotic coordination frameworks (ACFs), have been reported as an interesting approach to introduce new biochemical properties and enhance the activity of the drug taken.^9^ Well-known examples of metallodrugs are widely used in cancer treatment, such as platinum complexes cisplatin, carboplatin, or oxaliplatin.^9^

Metal complexes of quinolones have already been synthesized, favoring the coordination via the carbonyl and carboxylic/carboxylate groups and the metal center.^10^ Nonetheless, coordination by the β-keto acid moiety of quinolones to biocompatible metals is considerably attractive from a biopharmaceutical point of view. This hypothesis can be an alternative approach to obtain derivatives endowed with an improved antimicrobial activity, by manipulating the physicochemical characteristics of the resulting complex.^11^ Typical examples of application include complexes and ACFs of nalidixic and pipemidic acids with biocompatible metals such as zinc, magnesium, manganese, and calcium.^10−12^ It is important to note that these recent works have highlighted the increased attention toward the synthesis of complex active pharmaceutical ingredients (APIs) through solvent-free mechanochemical techniques, opening the way for the development of “medicinal mechanochemistry”.^13^ In particular, several of these new forms have been successfully prepared by mechanochemistry as a sustainable synthetic technique that has been applied in different fields, including coordination chemistry. It has proved to be an efficient, high-performance, and environmental-friendly as well as a clean and fast synthetic methodology to obtain potentially bioactive compounds in high purity and high (or even quantitative) yield, leading to significantly lower costs of production.^14^ In fact, the reaction is promoted by grinding together two or more compounds to induce the break/formation of covalent or supramolecular bonds, exploiting the energy derived from the grinding itself.^14^

To the best of our knowledge, there are limited works in the literature that focus on laboratory educational experiment approaches to medicinal mechanochemical synthesis. Typical examples of application include the synthesis of a small library of sulfonylureas with antidiabetic properties,^13^ the synthesis of polymers^15^ and the dual-drug naproxen-cimetidine coamorphous system.^16^ Though the preparation of Zn-nalidixic acid ACF was first reported in 2018,^12^ its synthesis, chemical characterization, and antibacterial activity evaluation as an educative approach are herein described for the first time.

## Experiment Overview

### Experiment Information

**Figure 2:**
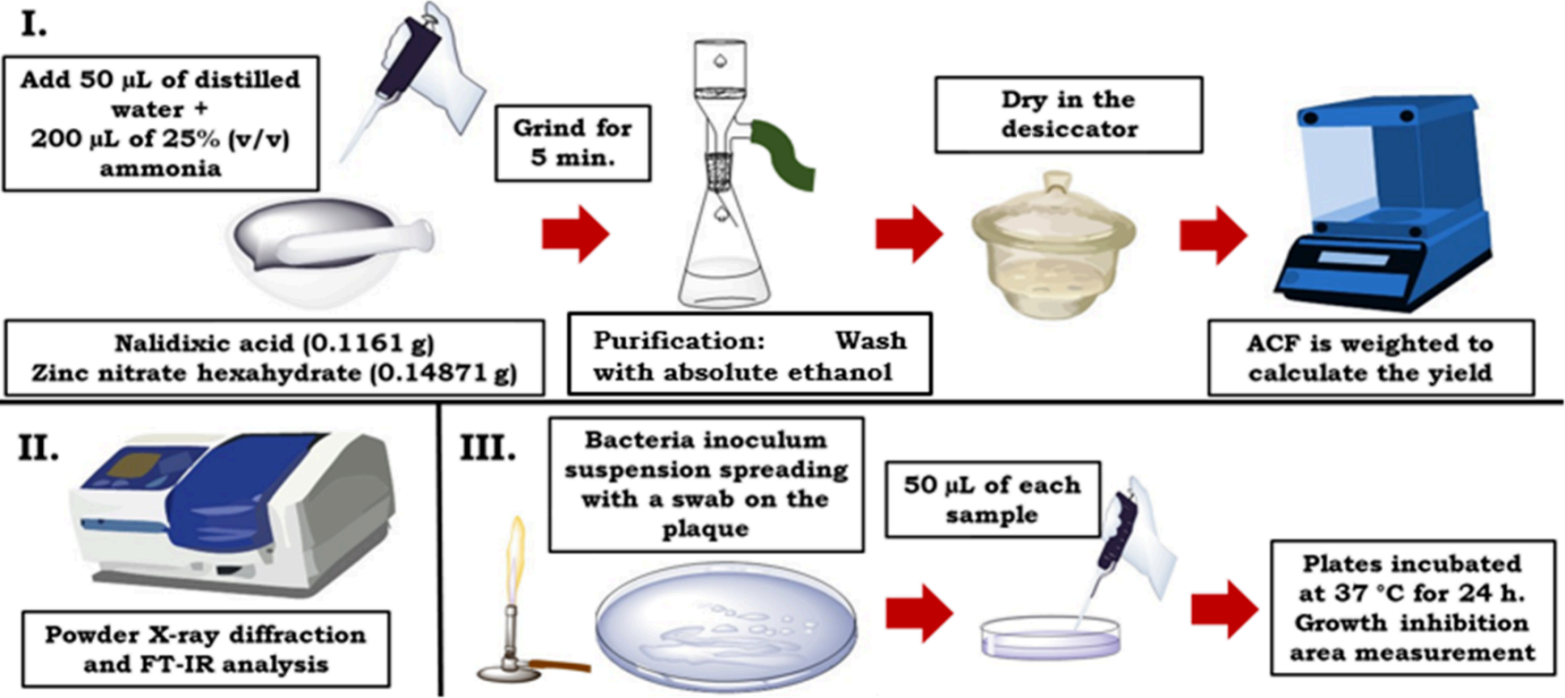
Schematic representation of the laboratory experiment. (I) Zn-ACF synthesis by mechanochemistry. (II) Structural characterization of Zn-ACF. (III) Antimicrobial evaluation of Zn-ACF.

**Figure 3:**
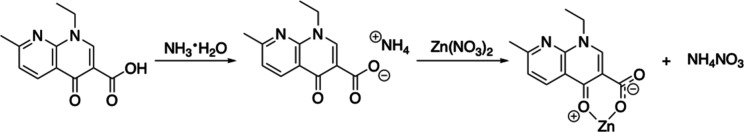
Reaction scheme for the synthesis of zinc nalidixic acid ACF.

A laboratory experiment for the subject of inorganic medicinal chemistry was taught to pharmacy and biochemistry students in their second year. The experiment focused on the importance of coordination chemistry and how it can be used as a tool to synthesize new metal complexes, starting by commonly available reagents (Figure 2). The students worked in groups of two or as individuals during 6 h, which were divided into three sessions. The first session introduces the students to the reaction of nalidixic acid and zinc nitrate, necessary to obtain the Zn-nalidixic acid ACF (Zn-ACF) (Figure 2.I). The Zn-ACF was synthesized by mechanochemistry (Figure 3), based on a procedure that was previously described.^12^ In particular, nalidixic acid and zinc nitrate hexahydrate were manually ground with distilled water in an aqueous ammonia solution by using a mortar and a pestle. The resulting solid was purified by washing with ethanol in order to remove the alcohol-soluble byproduct ammonium nitrate. The purity of the synthesized ACF was verified by TLC and carefully dried for the characterization step (see the Supporting Information for a detailed description).^17^

In the second session, the synthesized Zn-ACF was characterized by Fourier-transform infrared spectroscopy (FTIR) and powder X-ray diffraction (PXRD) (Figure 2.II) (see the Supporting Information for a detailed description). In the case of FTIR, a comparison with the spectrum of nalidixic acid highlighted the difference in the characteristic bands of the carbonyl and carboxyl groups involved in the coordination bond with the metal. As for PXRD, the samples were prepared and analyzed to initially check the formation of the Zn-ACF and the byproduct ammonium nitrate and finally confirm the presence of the pure desired ACF by comparing the resulting diffractograms with the predicted ones (see the Supporting Information for a detailed description).

Finally, in the third session, the antimicrobial activity of the obtained product was tested using the well-diffusion method and compared with that of the starting material, nalidixic acid (Figure 2.III) (see the Supporting Information for a detailed description). The Petri dishes, containing the solid agar medium, were prepared under septic conditions. *E. coli* was homogeneously inoculated, followed by the addition of Zn-ACF in dimethyl sulfoxide (DMSO), nalidixic acid (positive control), and DMSO (negative control) to each well in the medium (Figure 2.III). The growth inhibition area of each sample was measured in mm and compared to evaluate their antimicrobial activity on *E. coli* (see the Supporting Information for a detailed description). This test is extensively considered simple, highly reproducible, and cheap, and its data are easily interpretable with a good correlation to the standard reference guideline from the Clinical Laboratory Standards Institute (CLSI, formerly the National Committee for Clinical Laboratory Standards or NCCLS).^18,19^

For the prelab assessment (see the Supporting Information), the students were encouraged to read bibliographic information regarding the topics of synthesis, characterization, and bioactivity in order to prepare for the experimental activity. Additionally, students prepared a lab notebook with all important bibliographic information and procedures for the experiment. In all classes, the students’ lab notebooks were evaluated. The result of their experiments was reported in the lab notebooks. During the lab experiment, the students’ understanding of the experimental lesson was tested with postlab questions (see the Supporting Information). At the end of the course, the students’ final evaluation was conducted by preparing and presenting posters.

### Learning Objectives

The learning objectives of this experiment are to provide undergraduate students a multidisciplinary experience through the preparation, characterization, and antimicrobial evaluation of a nalidixic acid ACF. This will build a correlation between the didactic content and scientific research subjects. Upon completion of the laboratory experiment, students will be able to

- follow multistep procedures efficiently following the lab safety protocols;
- learn and apply a modern and sustainable methodological approach for the synthesis of novel bioactive entities, such as ACFs;
- understand the mechanism of formation of the Zn-nalidixic acid ACF by mechanochemistry;
- prepare samples of pure Zn-ACF and characterize them by FTIR and PXRD;
- interpret an FTIR spectrum, recognizing the characteristic bands of functional groups of interest, and compare it with the starting material to verify the formation of the product;
- analyze PXRD diffractograms and compare them with experimental or predicted patterns to verify the formation of the desired product and determine its purity;
- work under sterile conditions, essential for the reproducibility of microbiological assays;
- perform antimicrobial evaluation using the well-diffusion assay, measuring the growth inhibition zone and understanding its meaning in the presence of positive and negative controls;
- be able to present the results of the experience, using technical language, supported by a self-edited poster, showing how “soft skills” (such as speaking in public, corporal expression, creativity, etc.) can be easily developed in this framework.

## Hazards

During the experiments, laboratory coats, gloves, and goggles must be worn. Solvents must be placed and used in fume hoods. Undergraduates are required to consult the Material Safety Data Sheets (MSDSs) for all of the reagents associated with the chemicals used in this experiment. General information about the hazards of all relevant chemicals were provided. Nalidixic acid,^20^ zinc nitrate hexahydrate,^21^ ammonia,^22^ absolute ethanol,^23^ and DMSO^24^ can cause eye and skin irritation and are harmful if they are swallowed or inhaled. Nalidixic acid^20^ is suspected to cause fertility damage or damage to an unborn child, and it is fatal in the case of inhalation. Zinc nitrate hexahydrate can cause severe toxic effects after a single exposure.^21^ In the case of inhalation of any solvent, move to fresh air and seek medical attention. In the case of skin or eye contact with any of the reagents, one should be treated as described in the protocols and immediately seek medical attention.^20−23^ Ethanol^23^ and ethanol vapor are highly flammable. All reagents must be kept and stored away from heat, sparks, open flames, hot surfaces, and combustible materials.^25^ X-rays are highly energetic ionizing radiations and, as such, are potentially very hazardous to human health. Direct exposure to X-ray beams can lead to radiation damage, burnt skin and underlying tissue, and ultimately cancer and death; eye exposure to X-ray beams can lead to permanent cataracts and vision loss. Specifically, the part related to X-ray equipment is carried out by the teacher to prevent damage to the students. Therefore, it is important to follow all the safety rules detailed in the Supporting Information. *E. coli* (ATCC 25922)^26^ is a bacteria classified as biosafety level (BSL)-1 used to test the antimicrobial activity. BSL-1 bacteria present minimal potential hazard of infection, requiring a basic level of containment that relies on standard microbiological practices with no special primary or secondary barriers recommended.^27^ During the procedure, microbiology^28^ laboratory rules must be followed. The residues should be disposed of following the laboratory safety procedures to avoid release into the environment.^25^

## Results and Discussion

The synthesis of the Zn-ACF by the mechanochemistry methodology was reproduced by a typical lab session for second year pharmacy students as well as students in other health related fields, working in groups of two. The students were required to adequately grind solid nalidixic acid and zinc nitrate hexahydrate in the presence of aqueous ammonia. In this way, an external mechanical energy is given to the mixture of reagents by grinding them together with a mortar and a pestle. This energy is required from the system to convert the starting nalidixic acid to the carboxylate, which coordinates the zinc metal (Figure 3). After 5 min of grinding, the mixture was washed with a minimum amount of absolute ethanol, which removed the byproduct ammonium nitrate. The students synthesized the desired product with yields calculated from 10.8% to 82.4% (summary of results in Table S1). The observed wide range in terms of results could depend on the efficacy of the grinding step. If nalidixic acid does not completely react, only a small amount of insoluble product Zn-ACF can be obtained, since residual, unreacted nalidixic acid is washed out with ethanol, together with ammonium nitrate, and the purity was verified by TLC.

**Figure 4:**
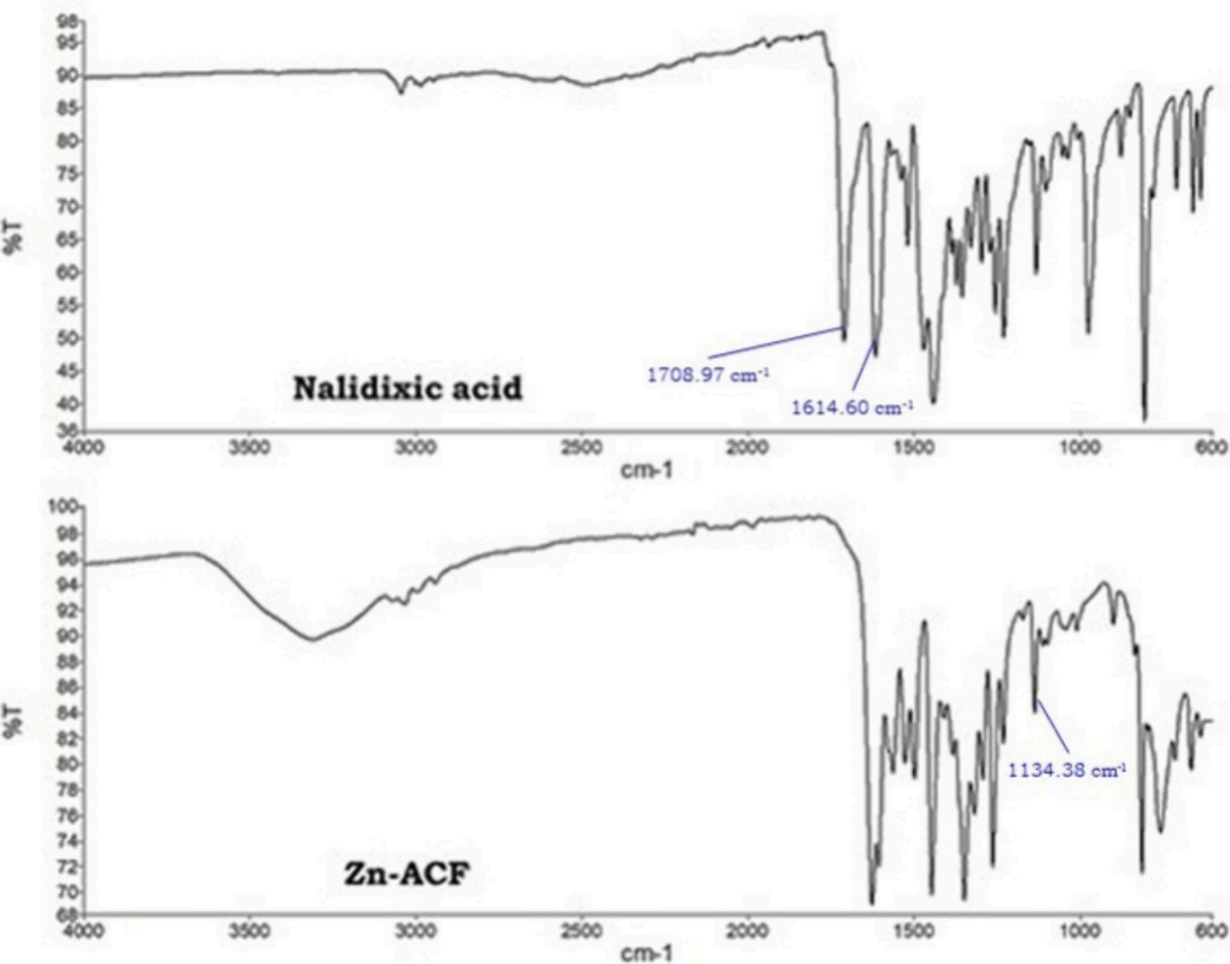
FTIR spectra of nalidixic acid (top) and Zn-ACF (bottom).

Once completely dried, FTIR analysis was carried out to verify the structure of the Zn-ACF, and the resulting spectrum was compared with that of nalidixic acid. Students learned how to use the FTIR equipment and put into practice what they learned during theoretical classes about this type of spectroscopic analysis. They found the implemented software to be very intuitive, and it was easy for them to efficiently interact with the equipment. A small amount of nalidixic acid and a solid sample were used for the acquisition, and the cleaning step in the analysis was paid attention. The resulting data were collected and carefully interpreted. From the spectra, two characteristic vibration bands of nalidixic acid were evident, the values of 1708.97 and 1614.60 cm^–1^ (Figures 4 and S2), corresponding to the stretches of the carboxyl and the carbonyl groups, respectively. In the spectrum of the complex Zn-ACF (Figures 4 and S3), the formation of the Zn–O bond was confirmed by the presence of a band at 1134.38 cm^–1^ (more details are in the Supporting Information).

**Figure 5:**
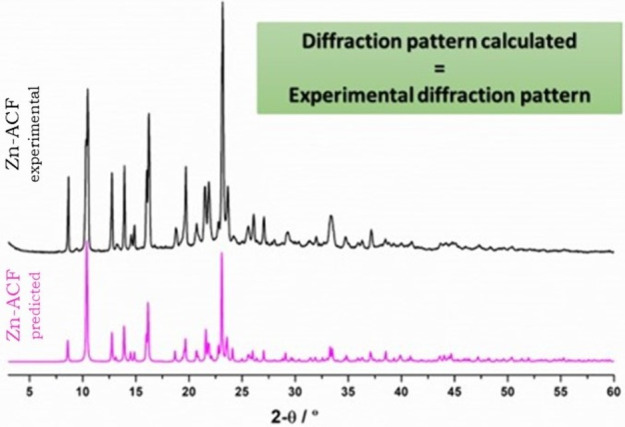
Comparison of the experimental PXRD pattern of the Zn-ACF (black) and the Zn-ACF predicted pattern (pink).

PXRD was used to confirm the formation of the Zn-ACF and its purity. Students learned how to prepare a sample for PXRD. Then, they were introduced to the equipment and the fundamental parameters that must be set for data collection. Finally, the collected data were analyzed. The comparison of the experimental diffraction PXRD pattern of the newly formed Zn-ACF (black diffractogram, Figure 5) with the Zn-ACF’s predicted pattern (pink diffractogram, Figure 5) revealed the same main peaks. It is possible to notice an overlap of the most significant peaks, with slight changes in terms of observed intensity, thus confirming the formation and purity of the intended Zn-ACF (more details are in the Supporting Information).

The antimicrobial activity of the Zn-ACF was evaluated against *E. coli* (Gram-negative bacteria) using the well-diffusion method. The students learned how to work under septic conditions, emphasizing that this aspect is very important while performing microbiological assays because of potential environmental contaminations. Several topics related to safely handling the bacteria were addressed. Students learned how to perform the assay, inoculate the Petri dishes, and prepare the samples at suitable concentrations in DMSO. The obtained results were expressed as growth inhibition area of each sample against *E. coli*. The students compared the inhibition zone of the Zn-ACF with that of nalidixic acid (positive control) and DMSO (negative control) (data are summarized in Table S2). All the groups carried out the experiment and obtained very similar results. Four out seven assays showed a growth inhibition area of the Zn-ACF > 11% higher than its precursor, nalidixic acid. In just one case, the difference was statistically irrelevant (G6, Table S2). The presence of DMSO did not interfere with the antimicrobial activity, as evidenced by the no-growth inhibition zone in the well of the negative control. Moreover, the students observed that the coordination of nalidixic acid to zinc resulted in relevant improvements in terms of antimicrobial profile.

Finally, the students presented the work that was carried out during a poster session for the final evaluation. The expected student learning outcomes for this experiment exceeded the expectations. The students were very interested and motivated in the preparation of their posters and were excited by the new form of oral communication that was incorporated into the course. The poster section was highly stimulating, and most of them actively participated in the discussion, making it an exchange of views about the practical experimental work and the theoretical part behind it. Moreover, this type of final evaluation process promoted the development of “soft skills” (such as public speaking, corporal expression, creativity, teamwork, leadership, community engagement, critical thinking, problem-solving, scientific writing, etc.^29^) and enhanced the students’ interest in the research area, not only in this particular experience. Overall, after all of the experiments, the students gained experience with laboratory techniques, such as filtration, used as a purification method, and chemical characterization aspects, while following the standard microbiological laboratory procedures. It was demonstrated that even noncovalent structural modifications could considerably influence biological activity. All these outcomes were addressed in pre- and postlab questionnaires to better evaluate the students’ performance during the experimental part. In general, the feedback was positive for all phases of the course.

In the future, it is expected that this laboratory experiment, at the interface between medicinal chemistry, microbiology, analytical techniques, public health, and pharmacology, will serve as a model for universities (or any other educational institution) to include mechanochemistry practices or other non-traditional sustainable synthetic methodologies in their curricula. Moreover, the project outcomes are going to be optimized by developing and implementing some service-learning practices. This will contribute to improving “soft skills” in the students, as they will be highly demanded by their future employers, in both business and academic fields.^30^ Specifically, service-learning deals with experiences designed to be mutual exchanges of knowledge and resources, aiming to promote academic and civic engagement as well as focus on holistic learner development and community well-being.^31^ Understanding the importance of antibiotic resistance and passing along the information to members of the community (projects that involved students in primary^29^ and high schools^32−35^) are other advantages of the reported exercise.

## Conclusion

Synthesis, characterization, and antimicrobial bioactivity of an ACF containing nalidixic acid and zinc, as an attempt to overcome multidrug resistance, were described for undergraduate teaching laboratories.

Students learned different topics, evaluated by prelab and postlab questions and a final evaluation, including mechanochemistry techniques and structural elucidation skills. The antimicrobial activities of the product ACF against *E. coli* were determined in the microbiology teaching lab, which will help students in their future careers.

## Supporting Information Available

Student handout containing an overview of the chemical and biological importance of nalidixic acid and the mechanochemical reaction, detailed experimental procedures, and pre- and postlab questionnaires; instructor notes (list of chemicals, equipment, hazards, safety information, instrumentation, and answers to the hints for student discussion); copies of the characterization data collected from student samples (PDF; DOCX)
