# Supplementary material for: Antibiotic Coordination Frameworks against Antibiotic Resistance: How to Involve Students through Experimental Practices in the Search for Solutions to Public Health Problems
Source: J Chem Educ. 2024 Mar 6;101(5):2045–51. doi: 10.1021/acs.jchemed.3c01125 (PMC11097387; doi:10.1021/acs.jchemed.3c01125)
Supplement: Supplementary file 1 — ed3c01125_si_001.pdf [file ed3c01125_si_001.pdf]

Antibiotic coordination frameworks (ACFs) against antibiotic resistance - How to involve students through experimental practices in the search for solutions to public health problems

Eva María Domínguez-Martín [1,2,†], Epole Ntungwe [1,2,†], Vera Isca [1,3,†], Salvatore Princiotto [4], Ana María Díaz-Lanza [2], Vânia André [5,*], Patrícia Rijo[1,3,*]

[1] CBIOS – Universidade Lusófona's Research Center for Biosciences & Health Technologies, Campo Grande 376, 1749-024 Lisbon, Portugal.
[2] Universidad de Alcalá de Henares. Facultad de Farmacia, Departamento de Ciencias Biomédicas (Área de Farmacología; Nuevos agentes antitumorales, Acción tóxica sobre células leucémicas. Ctra. Madrid-Barcelona km. 33,600 28805 Alcalá de Henares, Madrid, España.
[3] Instituto de Investigação do Medicamento (iMed.ULisboa), Faculdade de Farmácia, University of Lisbon, 1649-003 Lisbon, Portugal.
[4] Department of Food, Environmental and Nutritional Sciences (DeFENS), University of Milan, Via Celoria 2, Milan, 20133, Italy
[5] Centro de Química Estrutural, Instituto Superior Técnico, Universidade de Lisboa, Avenida Rovisco Pais, 1049-001 Lisbon, Portugal.
†These authors contributed equally to the study.
*Corresponding author: Prof. Dr. Patrícia Rijo (patricia.rijo@ulusofona.pt) and Prof. Dr. Vânia André (vaniandre@tecnico.ulisboa.pt)

TABLE OF CONTENTS

Instructor Notes .... **Error! Bookmark not defined.**

Lecture Before the lab experiment .... **Error! Bookmark not defined.**

1st Laboratory session: Chemistry (2 hours) .... **Error! Bookmark not defined.**-8

Introduction .... **Error! Bookmark not defined.**

Objective .... **Error! Bookmark not defined.**

List of Equipment and Chemicals .... **Error! Bookmark not defined.**

Hazard and safety .... **Error! Bookmark not defined.**

General Remarks.. **Error! Bookmark not defined.**

Pre-lab assesment .... **Error! Bookmark not defined.**

Experimental procedure .... **Error! Bookmark not defined.**

Experiments reproduction and students results .... **Error! Bookmark not defined.**

Experiment's Photos 7

Post-lab assessment 7-8

Instructor notes for the experiments........................ **Error! Bookmark not defined.**

2nd Laboratory session: Structural Characterization (2 hours) ..... **Error! Bookmark not defined.**-15

Introduction .... **Error! Bookmark not defined.**

Objective .... **Error! Bookmark not defined.**

List of Equipment and Chemicals 9

Hazard and safety .... **Error! Bookmark not defined.**

Pre-lab assesment 10

Experimental procedure .... **Error! Bookmark not defined.**

Experiments reproduction and students results .. **Error! Bookmark not defined.**-14

Pos-lab assessment .... **Error! Bookmark not defined.**-15

Instructor notes for the experiments........................ **Error! Bookmark not defined.**

3rd Laboratory session: Microbiology (2 hours) .... **Error! Bookmark not defined.**-19

Introduction .... **Error! Bookmark not defined.**

Objective .... **Error! Bookmark not defined.**

List of Equipment and Chemicals .... **Error! Bookmark not defined.**-16

Hazard and safety .... **Error! Bookmark not defined.**
Pre-lab assesment .... **Error! Bookmark not defined.**
Experimental procedure .... **Error! Bookmark not defined.**
Experiments reproduction and students results .... **Error! Bookmark not defined.**
Pos-lab assessment .... **Error! Bookmark not defined.**
Instructor notes for the experiments................... **Error! Bookmark not defined.**-20
References..20-21

Instructor Notes

This supplementary information was prepared to be a guideline to teach the synthesis, characterization, and antimicrobial evaluation of a bioactive organometallic complex (ACF) to students. This material is intended as a resource for pharmacy/biochemistry/chemistry students in subjects related to medicinal inorganic chemistry.

Students should fulfil the experiment in groups of 2-3 depending on class size. The experiment is divided into three parts, divided into classes of two hours per week. Each group should fulfil all the tasks in all the classes in 2h. For the last class (microbiology evaluation), the experiment should be completed by the students in 2 hours; however, the bacteria need to be incubated for 24 h. For this reason, the students are recruited for the last session after 24 h to analyse the results (about 10 minutes for each group).

Lecture Before the lab experiment

Before the lab experiment, the lab instructor should present the experiment and discuss the main goals of each task and how to achieve them. The concept of mechanochemistry should be explained, and the role of each reagent should be highlighted. Fourier-transform infrared spectroscopy (FTIR) and powder X-ray diffraction (PXRD) concepts should be addressed, such as equipments' operativity, security issues (PXRD), and expected outcome (spectra and diffractograms, respectively). Additionally, several topics related to the safe handling of bacteria should be addressed. Students should be familiar with the concept of sterile environment, bacteria inoculation, and growth inhibition. Explain to students how to prepare the samples at the requested concentrations.

1st Laboratory session: Chemistry (2 hours)

Introduction

Antibiotic resistance is a global concern of the 21st century. Therefore, making chemical improvements on classic antibiotics can be a strategy to overcome this problem. In this context, the present work proposes the synthesis of an ACF comprising nalidixic acid (quinolone) and Zinc, through mechanochemistry.

Mechanochemistry is an emergent sustainable synthetic technique that has been applied in coordination chemistry. It consists of grinding together two or more compounds to promote a reaction, by inducing the breaking/formation of covalent or supramolecular bonds, exploiting the energy developed from the grinding itself. It is an efficient, high-performance, environmental-friendly, clean, and fast synthetic procedure by which the desired products are obtained in high purity and yield.[1]

Objective

- Synthesize a bioactive organometallic complex Zn-ACF from nalidixic acid and Zinc by mechanochemistry.

List of Equipment and Chemicals

- **Equipment and instrument**

▪ Analytical scale	▪ Measuring cylinder 20 mL
▪ Weighing paper or glass	▪ Büchner funnel
▪ Spatulas	▪ Filter paper
▪ Micropipette p50 and p200	▪ Kitasato flask
▪ Mortar and pestle	▪ Desiccator

- **Reagents and solvents**

All reagents used are commercially available in Sigma-Aldrich, Panreac, Merck, and VWR International and have been used without further purification.

Name	CAS
▪ Nalidixic acid (Sigma-Aldrich)	CAS (389-08-2)
▪ Zinc nitrate hexahydrate (Merck)	CAS (10196-18-6)
▪ Distilled water	CAS (7732-18-5)
▪ Absolute ethanol	CAS (64-17-5)
▪ 25% (v/v) aqueous ammonia solution (Merck)	CAS (1336-21-6)

Hazard and safety

During all the steps, use lab coat and safety goggles. The reagents must be used in the fume hood, kept and stored away from heat, sparks, open flames, hot surfaces, and combustible materials. Do not smoke. Do not release the chemical compounds into the environment. In case of inhalation of any solvent, inhale fresh air and seek medical attention. Areas of skin or eye contact should be treated as described in the protocols and immediately, looked for medical attention.[2-7]

- **Nalidixic acid:** Hazardous if swallowed and in case of skin contact. Suspected of damaging fertility and the unborn child (Reproductive toxicity). Fatal if inhaled.[3]
- **Zinc nitrate hexahydrate:** Harmful if swallowed, inhaled (irritation), in case of dermal contact (irritation, corrosion), in case of eye contact (serious damage/eye irritation). Specific target organ toxicity, single exposure.[2]
- **Distilled water:** None.
- **25% (v/v) Ammonia:** Harmful if dermal contact (causes severe skin burns), eye contact (damage), and/or inhaled (irritation of the respiratory tract).[4]

- **Absolute ethanol:** Hazardous in case of skin contact (toxic irritant and permeator), eye contact (serious irritation), of inhalation, and ingestion (it may cause damage to organs). Highly flammable liquid and vapor.[5]

Pre-lab assessment

Students' notebook: the experiment should be previously prepared in the notebook. It should include the following information:

a. Introduction
b. Material and reagents
c. Important safety data information
d. Experimental procedure (written or in scheme)
e. References

Experimental procedure

1) Weigh 0.1161 g of nalidixic acid and 0.14874 g of zinc nitrate hexahydrate ($Zn(NO_3)_2{\cdot}6H_2O$) on the analytical balance.
2) Add 50 μL of distilled water and 200 μL of 25% (v/v) aqueous ammonia solution to the previous mixture.
3) Grind the mixture of the reagents with the aid of a mortar and a pestle for approximately 5 minutes (**Note 1**).
4) Wash the bulk product with absolute ethanol, under reduced pressure, to remove the by-product (NH_4NO_3) (using the minimum amount possible of solvent).
5) Allow to dry in the desiccator and weigh on the analytical balance (calculate the yield).

Note 1. A small quantity of product is obtained (see photo **FS1.III. and IV.**).

Experiment reproduction and students' results

Students obtained the product Zn-nalidixic acid ACF (Zn-ACF) as an amorphous white solid in yields ranging from 10.8 to 82.4% (**Table S 1**). The weight of nalidixic acid used for the reaction and the wight of the final product of each group are summarized in **Table S 1**.

Table S 1: Results from the students' experiments using previously optimized conditions.

Groups	Nalidixic Acid weight (g)	Zn-ACF weight (g)	Yield (%)
**G1**	0.1158	0.0843	56.8
**G2**	0.1174	0.0337	22.4
**G3**	0.1161	0.0348	18.3
**G4**	0.1171	0.1237	82.4

**G5**	0.1175	0.0162	10.8
**G6**	0.1172	0.04	26.6
**G7**	0.1165	0.04	26.8

Experiment's Photos

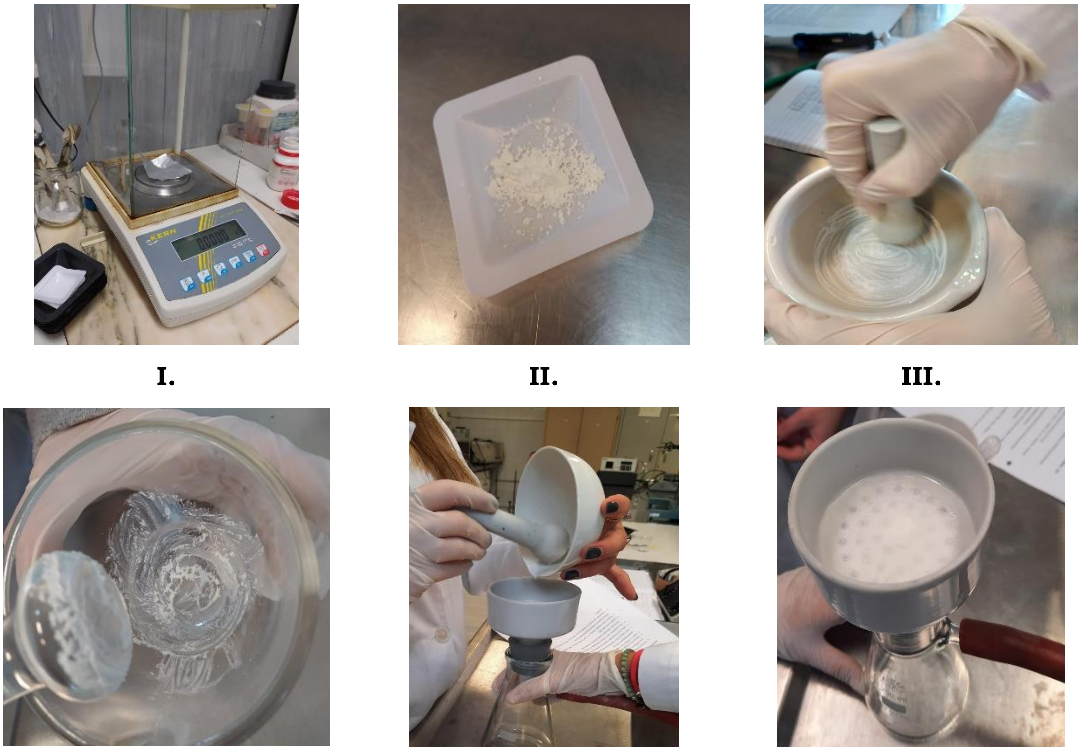

I. **II.** **III.**

IV. **V.** **VI.**

Figure S1: Experimental procedure' photos: I. and II. Weighing; III. and IV Mixture grinding; V. and VI. Filtration of the product.

Post-lab assessment

- Indicate the objective(s) of this first class.

Answer: The objective of this lab experience is to synthesize a bioactive metalorganic complex starting from nalidixic acid and zinc by mechanochemistry.

- Indicate the method used to accomplish the objective.

Answer: The method used to accomplish the first objective was the synthesis by mechanochemistry.

- Explain and describe some advantages of the method used.

Answer: Mechanochemistry is a sustainable synthetic technique that deeply minimizes the use of solvents, leading to the formation of new compounds in a fast and efficient way.

- Calculate the obtained yield.

Answer: $\eta\ (\%) = \frac{m\ obtained}{m\ teoretical} \times 100$,

where: $m\ teoretical = \frac{M\ [ACF] \times m\ Nalidixic\ Acid}{M\ [Nalidixic\ Acid]}$

Example: For the measured mass of 0.1161 g of nalidixic acid and the obtained mass of Zn-ACF of 0.1209 g:

$m_{\text{ theoretical}} = \frac{297.63 \times 0.1161}{232.24} = 0.1488$ g

$\eta\ (\%) = \frac{0.1209}{0.1488} \times 100 = 81.3\ \%$

Instructor notes for the experiments

- The trickiest step of this class is the wash of the bulk product, under reduced pressure. The wash should be done to eliminate the by-product NH_4NO_3. Special attention should be paid to the amount of solvent used. A minimum amount of solvent should be used to minimize the dissolution of the Zn-ACF, and consequent loss of yield.
- If the students obtain a yield higher than 100%, probably the product is not completely dry, or the salt was not adequately washed out.
- At the end of this step, the students are expected to understand mechanochemistry as a suitable alternative for the synthesis of the compound of interest.

2nd Laboratory session: Structural Characterization (2 hours)

Introduction

FTIR is a technique used to obtain an infrared spectrum of absorption or emission of a sample. Every molecule has its peculiar vibrational behavior and it can be distinguished from that of other molecules. Thus, the infrared spectrum is characteristic for each molecule and can be used to study and identify molecules or functional groups within the molecules. The information that can be deduced from infrared spectrum is complementary to that of other methods.[8]

PXRD analysis is a rapid analytical methodology used for the characterization of solid compounds. This technique measures the diffraction pattern of a crystalline material as a powder of the material, rather than an individual crystal. Powder diffraction is often easier and more convenient than single-crystal diffraction since it does not require individual crystals to be made. The analysed material is finely ground, homogenized, and the average bulk composition is determined. Diffraction peaks observed in the PXRD pattern for a crystalline product are unique.[9]

Objective

- Characterize the product obtained in the previous class.

List of Equipment and Chemicals

- **Equipment and instrument**

▪ FTIR

The FTIR spectra were obtained using the PerkinElmer® Spectrum 400 (PerkinElmer Inc, Waltham, MA, USA) equipped with an attenuated total reflectance (ATR) device.

▪ PXRD

PXRD diffraction data were collected with D8 Advance Bruker AXS θ-2θ diffractometer, with a copper radiation source (Cu Kα, λ = 1.5406 Å) and a secondary monochromator, operated at 40 kV and 40 mA. Note that a Ni filter was not used in the data collections, and therefore the k-β peak at around ~9° in 2-θ is present in the experimental diffractograms.

- **Reagents and solvents**

Name	CAS
▪ Distilled water	CAS (7732-18-5)
▪ Absolute ethanol	CAS (64-17-5)
▪ Mixture water/ethanol (50:50 v/v)	

Hazard and safety

During all the steps, use a lab coat and safety goggles. The reagent must be used in the fume hood. Absolute ethanol is hazardous in case of skin contact (toxic irritant and permeator), of eye contact (serious irritation), of inhalation, and ingestion (it may cause damage to organs). Highly flammable liquid and vapor.[5]

X-Ray radiation is a highly energetic ionizing radiation and, as such, is potentially very hazardous to human health. Direct exposure to X-ray beams can lead to radiation damage, burnt skin and underlying tissue, and ultimately cancer and death; eye exposure to X-ray beams can lead to permanent cataracts and loss of vision. So, it is important to follow all the safety rules:

1) Only trained personnel are permitted to operate the diffractometers. Specifically, this part by the teacher with expertise with the equipment to prevent damage to students.
2) Strictly follow all the safety protocols for each piece of equipment.
3) Always confirm that all the exposures are completed, and the shutter is closed before opening the lead doors and accessing the X-ray enclosure.
4) Never bypass the safety interlocks for any reason.
5) Close the enclosure lead doors before starting data collection.

Pre-lab assessment

Students' notebook: the experiment should be previously prepared in the notebook. It should include all the information of the first class, including the obtained results and the following information about the second class:

a. Material and reagents
b. Important safety data information
c. Experimental procedure (written or in scheme)
d. References

Experimental procedure

1) Preparation of the acquisition on the FTIR program: background scanning, setting sample' name and directory to store.
2) Placing a small portion of the solid previous obtained in the diamond in the equipment.
3) Spinning the screw into the sample (pre-view the spectrum in the program to set the pressure to apply before starting the acquisition).
4) The cleaning of the equipment is done with the mixture of water/ethanol (50:50 v/v).
5) Repeat steps 1-5 for the analysis of nalidixic acid.
6) Characterization of the synthesized Zn-ACF by comparison of the FTIR spectra obtained.
7) Preparation of a sample of the synthesized Zn-ACF for PXRD.
8) Definition (with the help of the tutor) of the best program to collect PXRD data for the bulk sample.
9) Acquisition of PXRD data for the Zn-ACF.
10) Demonstration of how to generate the theoretical PXRD pattern corresponding to the structure.
11) Comparison between the diffractogram of ACF and the theoretical diffraction pattern, in order to ensure the bulk's purity.
12) Comparison between the diffraction patterns of the starting materials (Nalidixic acid and zinc nitrate 6-hexahydrate) and the product, to verify the formation of the new compound.
13) Identification of NH_4NO_3 as by-product by searching within the Crystallography Open Database (COD).

Experiments reproduction and students' results

FTIR and PXRD analysis were used to confirm the structure of the Zn-ACF complex synthesized in the first class.

- **FTIR Results**

The infrared spectrum is unique to each structure and similar spectra were obtained for all the groups, confirming that they all obtained the same final product.

Figure S2 shows the infrared spectrum of the sample of pure nalidixic acid. Aryl carboxylic acids absorb at about 1725-1700 cm^{-1} due to C=O carbonyl stretching. Strong bands at 1708.97 and 1614.60 and cm^{-1} are characteristic of the stretching vibrations of the C=O group of carboxylic acid and pyridone, respectively. The weak and broadband at 2487.51 cm^{-1} is assigned to the carboxylic acid O-H stretching vibration. Aromatic hydrocarbons show absorption in the region of 1440 cm^{-1} due to C=C symmetric stretching vibrations in the aromatic ring. Absorption peaks at 1517.37, 1468.03, and 1293.94 cm^{-1} are assigned to the deformation vibration of the Nitrogen-carbon-hydrogen (NCH). Interactions between C=N stretching vibration result in three strong to medium absorptions at 1383.93, 1468.03, and 1517.37 cm^{-1}. The weak bands at about 3044.19–2950 cm^{-1} are assigned to aliphatic CH stretching vibration of ethyl and methyl groups present in nalidixic acid.[10-12]

The FTIR spectrum of the Zn-ACF (**Figure S3**) shows a very strong peak at 1624.44 cm^{-1} attributed to the C=O stretching vibration of the pyridone. The shift of approximately 10 cm^{-1} for the C=O stretching vibration of the pyridone compared to the nalidixic acid (detected at 1614.60 cm^{-1}), suggested that coordination occurs via the β-keto acid portion. The characteristic stretching vibration of the COO at 1708.97 cm^{-1} in the nalidixic acid (**Figure S2**) is not detected in the Zn-ACF FTIR spectrum (**Figure S3**). This result indicates that COO group in Zn-ACF has a negatively charged oxygen atom. The asymmetric stretching bands of the COO^- group of the ACF is strongly detected at 1605.37 cm^{-1}. The stretch of the Zn-O bond is detected at 1134.38 cm^{-1}. The broad band found in the 3000-3600 cm^{-1} region is representative of the hydrogen bonds found in this structure, as well as the stretching vibration of the coordinated water molecules.[3]

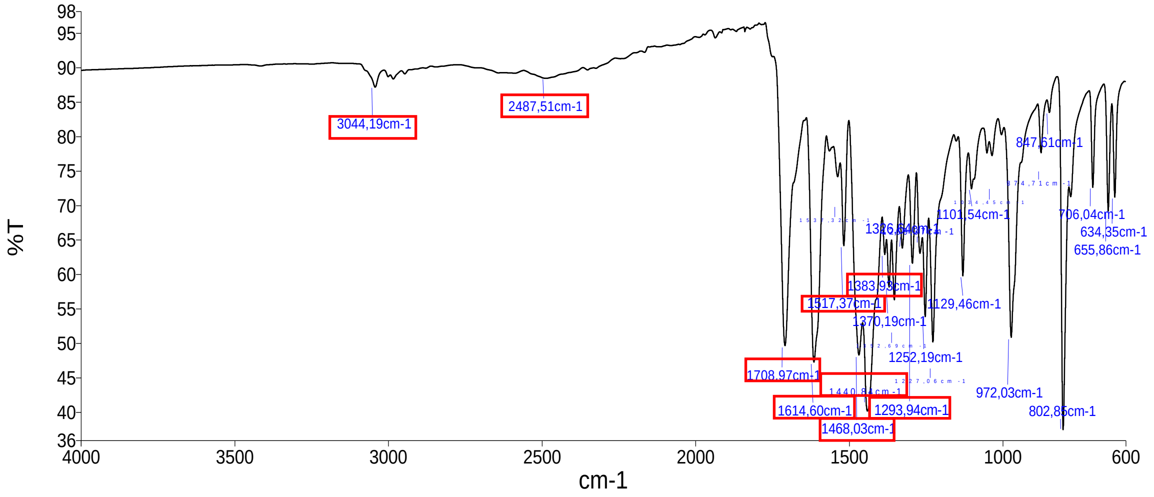

Figure S2. FTIR spectrum of Nalidixic Acid.

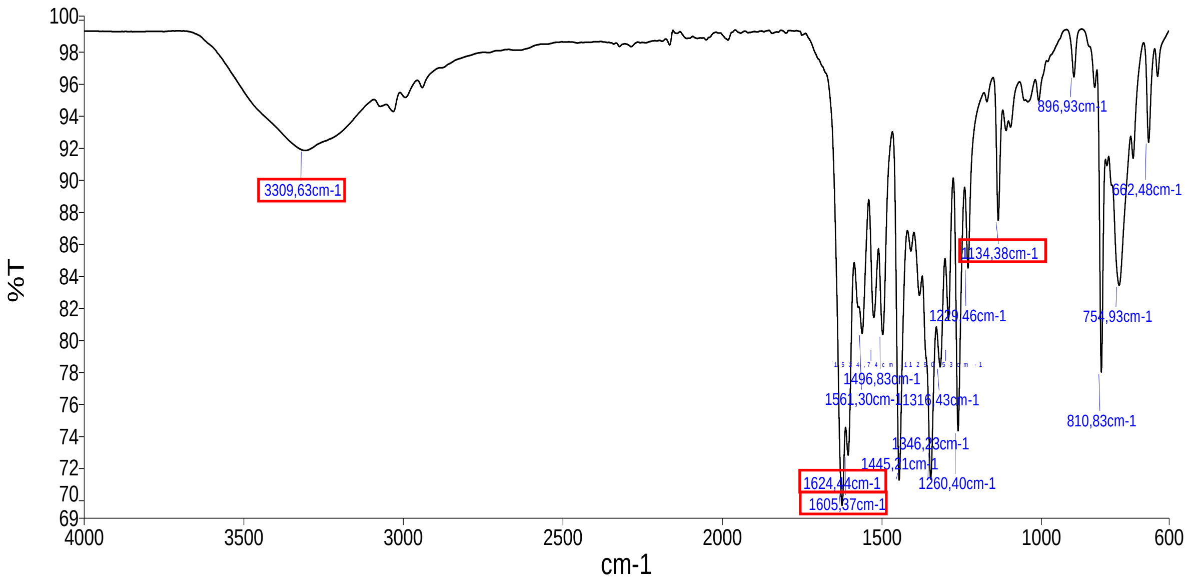

Figure S3. FTIR spectrum of Zn-ACF.

- **Powder X-ray diffraction data**

Figure S4 shows the PXRD pattern of the newly synthesized Zn-ACF. The diffraction pattern is unique to each compound and similar PXRD patterns were obtained for all the groups, confirming that they all obtained the same final product.

Nalidixic acid diffraction pattern can be experimentally obtained from the starting material, or it can be simulated from the data available at the Cambridge Structural Database. The comparison of diffraction patterns of nalidixic acid and the obtained powder, reveal significant differences, as seen in **Figure S5**. This proves that the reaction occurred, and a new product was obtained. Moreover, comparing the experimental PXRD pattern of the powder with the Zn-ACF predicted pattern obtained from the crystal structure data, it is possible to notice an overlap of the peaks, confirming the formation of the intended Zn-ACF.

Furthermore, in **Figure S6** it is possible to observe the experimental PXRD pattern of the Zn-ACF before (pink) and after being washed (blue), the Zn-ACF predicted pattern (black) and the NH_4NO_3 peaks (red, obtained from the COD). The NH_4NO_3 peaks (red, **Figure S6**) can be also identify in the PXRD pattern of Zn-ACF before being washed (pink **Figure S6**), proving the formation of the by-product in the mechanochemistry reaction. After washing, (blue, **Figure S6**) the referred peaks are not present anymore, supporting the elimination of the by-product in the washing step. In summary, it was shown how PXRD is used to assess the formation of a new compound, by comparing the diffraction pattern of the newly formed product with the those of the starting material and prediction. The technique was further used to confirm the formation of NH_4NO_3 as a by-product. PXRD data also confirms the purity of the bulk.

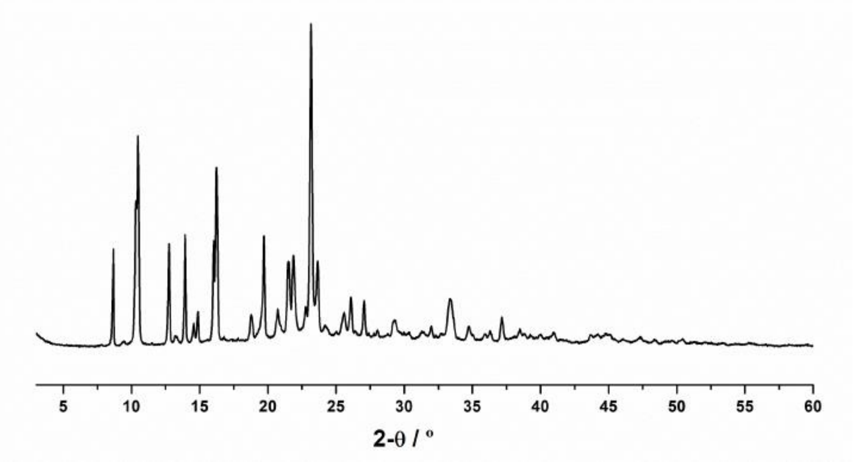

Figure S4. PXRD pattern of the final pure bulk sample analysed in the class. All groups reached a similar result, with the 2-θ values being similar for all.

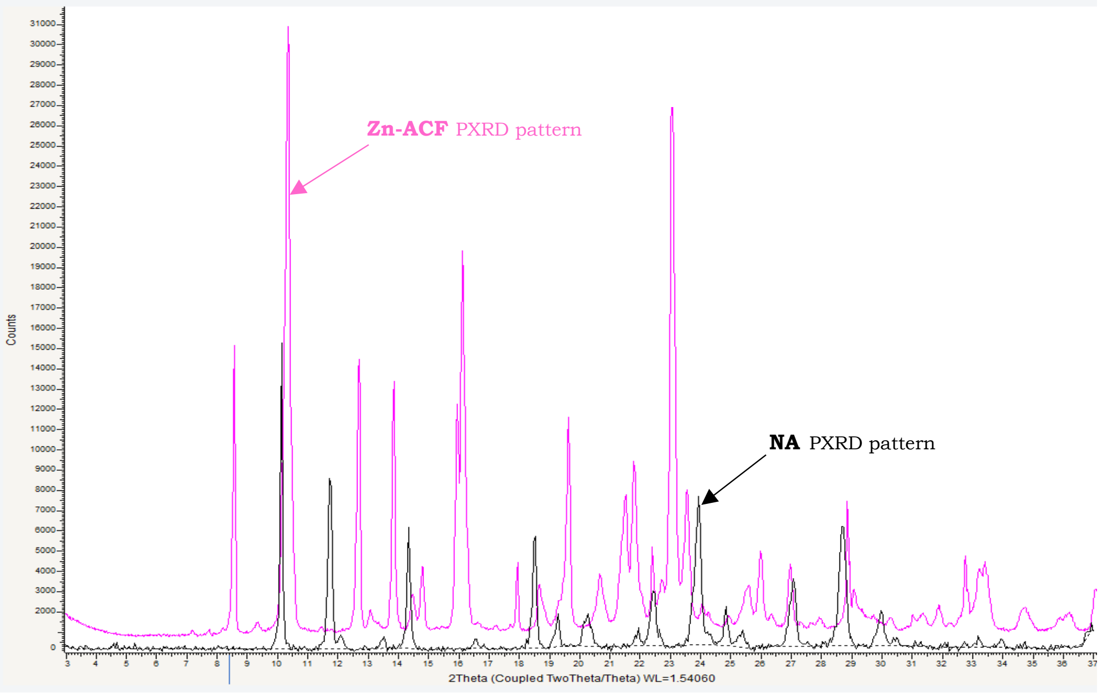

Figure S5. Comparison of the experimental PXRD pattern of the newly formed Zn-ACF (pink) with nalidixic acid's pattern (black) to assess the formation of a new product.

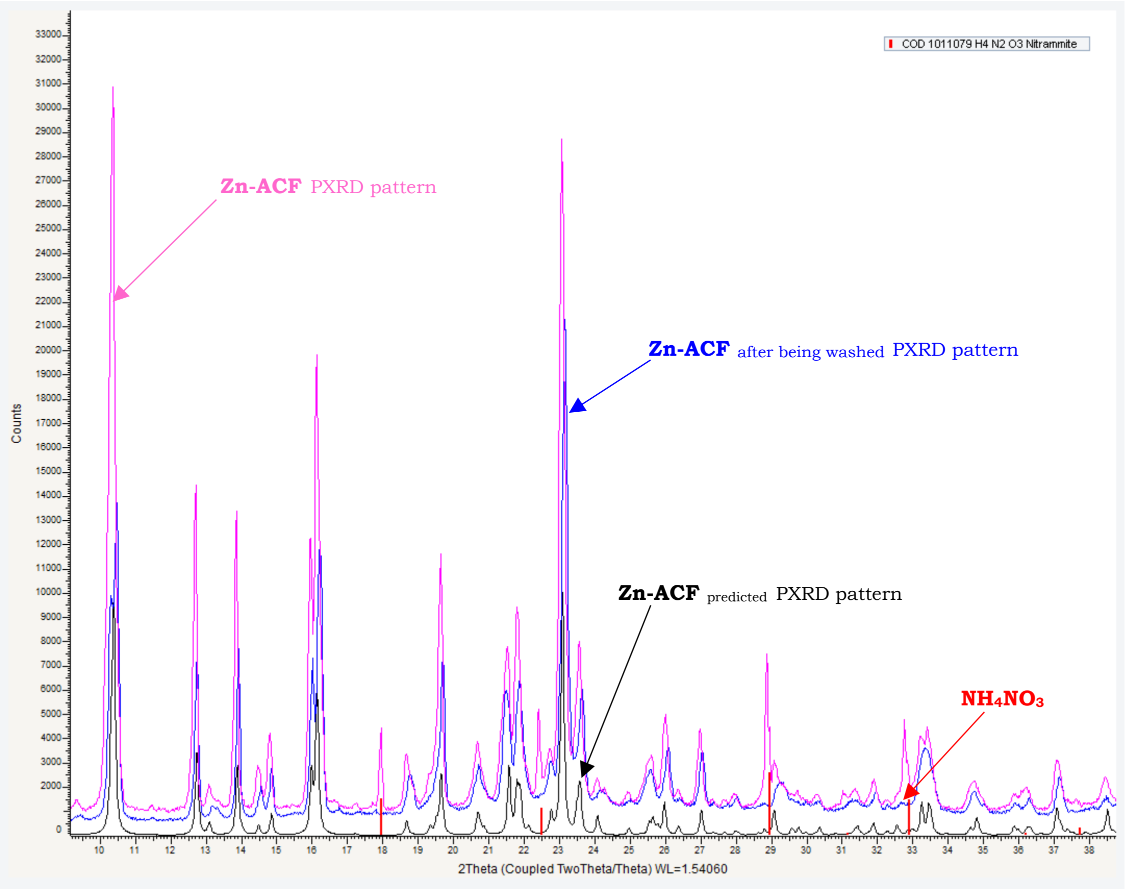

Figure S6. Comparison of the experimental PXRD pattern of the newly formed Zn-ACF before (pink) and after being washed (blue) compared with the Zn-ACF's predicted pattern (black) and the NH_4NO_3 peaks (red).

Post-lab assessment

- Indicate the objective(s) of this class.

Answer: This experimental work aims to confirm the formation of the Zn-ACF complex by mechanochemistry.

- Indicate the methods used for the characterization of the formed complex. Explain the purpose of each technique used.

Answer: Methods used for the characterization of the formed complex include:

- ✓ FTIR analysis: obtain an infrared spectrum sample. The infrared spectrum is unique for each molecule and can be used to study and identify molecules or functional groups within the molecules.
- ✓ PXRD analysis: rapid analytical methodology used for the characterization of solid compounds. This technique measures the diffraction pattern of a crystalline material and the observed diffraction peaks are unique for each product.

- How can PXRD be useful?

Answer: PXRD data can be used to 1) follow the reactions and determine the formation of new products; 2) phase identification, with identification of possible by-products; 3) check the purity of the final product; 4) follow the shelf life of the product over time.

Instructor notes for the experiments

- At the end of this step, the students are expected to understand the importance of the techniques used for the structural characterization.
- Students should understand that each FTIR spectrum is characteristic for each structure, due to the characteristic stretching vibration of each group present in the molecules.
- Students should understand how PXRD is used to assess the formation of a new compound and its purity.

3rd Laboratory session: Microbiology (2 hours)

Introduction

The introduction of effective antibacterial therapies for infectious diseases has completely revolutionized clinical practices.[13] Nonetheless, antibiotic resistance is a global concern of the 21st century. Therefore, making chemical improvements on classic antibiotics can be a strategy to overcome this problem. In this context, the newly prepared Zn-ACF complex is being evaluated as antimicrobial agent. Once nalidixic acid has proven activity against gram-negative bacteria,[14] the gram-negative *Escherichia coli* (ATCC 25922) was selected for this experiment. The microbial activity will be evaluated by the well-diffusion method. In this method, agar plates are inoculated with a standardized inoculum of the test microorganism. Generally, the antimicrobial agent diffuses into the agar and inhibits germination and growth of the test microorganism. This method is widely used to evaluate the antimicrobial activity because it is rapid and inexpensive. Thus, it is very useful and efficient as screening tool.[15]

Objective

- Antimicrobial activity of nalidixic acid and Zn-ACF complex.

List of Equipment and Chemicals

- **Equipment and instrument**

▪ Bunsen burner	▪ Micropipettes tips
▪ Sterile loops (wire/plastic)	▪ Glass Pasteur pipettes

▪ Sterile cotton swabs	▪ Incubator
▪ 0.5 McFarland standard	▪ Marker pen
▪ Micropipettes (p200 and p1000)	▪ Plastic ruler
▪ Mueller-Hinton plates (Biokar Diagnostics, Beauvais, France).	

- **Reagents and solvents**

Name	CAS
▪ Ethanol 70%	CAS (64-17-5)
▪ Nalidixic Acid	CAS (389-08-2)
▪ Dimethyl sulfoxide	CAS (67-68-5)
▪ Sterile water	
▪ Synthetized Zn-ACF	

- **Microorganism**

Escherichia coli (ATCC 25922)

Hazard and safety

Use protective clothing, gloves, and safety goggles.

- **Nalidixic acid** CAS (389-08-2): Hazardous if swallowed and in case of skin contact. Suspected of damaging fertility or the unborn child (Reproductive toxicity). Fatal if inhalation.[3]
- **Ethanol** CAS (64-17-5): Hazardous in case of skin contact (toxic irritant and permeator), of eye contact (serious irritation), of inhalation, and ingestion (it may cause damage to organs). Highly flammable liquid and vapor.[5]
- **Dimethyl sulfoxide** CAS (67-68-5): Irritating to the skin, eyes, nose, throat, and respiratory tract.[16]
- ***Escherichia coli*** (ATCC 25922): is a bacteria classified as biosafety level (BSL) 1 used to test the antimicrobial activity.[16] BSL-1 bacteria present minimal potential hazard of infection to laboratory personnel and the environment and should be handled under BSL-1 guidelines from the Center for Disease Control.[17]

According to the Institutional Biosafety Committee (IBC) advises on matters relating to the safe handling, transport, use, and disposal of biological materials, the readers of this experiment should consult the Required Training for work at Biosafety Level 1 (https://www.drs.illinois.edu/Programs/RegistrationInformation#RequiredTraining).[18]

Pre-lab assessment

Students' notebook: the experiment should be previously prepared in the notebook. It should include all the information of the first two experiments and the following information about the second class:

a. Material and reagents
b. Important safety data information
c. Experimental procedure (written or in scheme)
d. References

Experimental procedure [15]

1) Preparation of sample solutions:
   a) Weight a small amount (around 5-15 mg) of each compound (Zn-ACF and nalidixic acid) in Eppendorf tubes (or other vials) and dissolve them in dimethyl sulfoxide (DMSO), to reach the final concentration of 10 mg/mL (e.g.: 970 µL for 9.7 mg to achieve 10 mg/mL).
   b) Dilute the previous solution to 1 mg/ml (e.g.: 100 µL of the previous solutions and dilute them with 900 µL of DMSO to achieve 1 mg/mL - 1:10 dilution in DMSO).
2) To create aseptic conditions, clean the worktable with 70% alcohol (v/v), let it dry, and turn on the Bunsen burner.
3) With a sterile cotton swab, dip into the inoculum of the microorganism (*E. coli* (ATCC 25922)) and pass it through the Petri dish containing its solid culture medium (agar Mueller-Hinton) **(Note 1)**.
4) Repeat the process at least 5-6 times to ensure that the microorganism is homogeneously distributed.
5) Wells (5 mm) were created into the solid medium using a sterile glass Pasteur pipette.
6) Identify each well with a sample (compound prepared in the mini-project class), positive control, and negative control.
7) Add 50 µL of the test compounds to diffuse, the positive control (nalidixic acid), and the negative control (DMSO) at a concentration of 1 mg/mL in DMSO to each respective well.
8) Label the plates and place them in the oven at 37°C. Incubate for 24 h. Perform all the tests in triplicate.
9) After incubation using a ruler, calliper, or an automated zone reader measure the diameter in millimetres (mm) of the zone of inhibition of the tested compounds.

10) Read the plates from the back against a dark background illuminated with reflected light.
11) Evaluate the inhibition area of each compound against the bacteria strain *E. coli.* Compare the antimicrobial activity of nalidixic acid and Zn-ACF.

Note 1: The microbial suspensions should be previously prepared by the instructors.

Experiments reproduction and students' results

The antimicrobial activity of nalidixic acid and Zn-ACF samples against *E. coli* was evaluated by the students using the well diffusion method (DMSO was used as negative control). The obtained results are presented in **Table S 2** and showed an increase of the growth inhibition area of Zn-ACF complex comparing to the starting material (nalidixic acid) (**Figure S13**). Higher inhibition area denotes higher antimicrobial activity. Thus, results suggest that the structural modification present in the Zn-ACF increased the nalidixic acid antimicrobial potential against *E. coli* strain.

Table S 2: Antimicrobial activity results on *E. coli* using the Well Diffusion Test. Diameter of Inhibition zone (mm).

	Growth inhibition area (mm)	
**Groups**	**Zn-ACF**	**Nalidixic acid (NA)**
**G1**	28	25
**G2**	28	26
**G3**	29	26
**G4**	28	26
**G5**	28	24
**G6**	27	26
**G7**	28	25

DMSO = negative control, 5 mm of growth inhibition zone

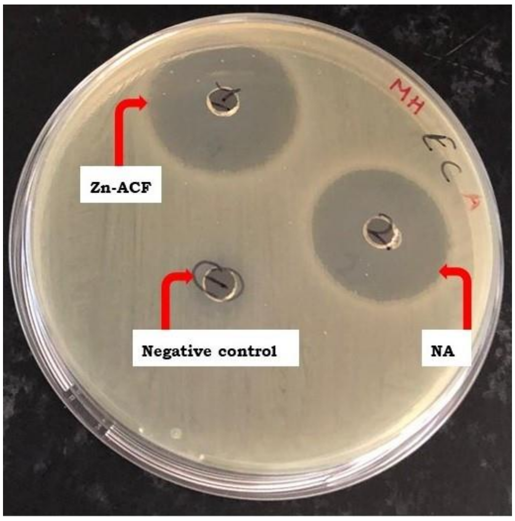

Figure S13. Plate after 24 h incubation at 37 °C.

Post-lab assessment

- Indicate the objective(s) of this class.

Answer: This experimental work aims to evaluate the antimicrobial activity of both the obtained complex and the starting material.

- Indicate the method used for antimicrobial activity evaluation. Explain the method and highlight one advantage.

Answer: The method used was the well-diffusion method. In this method agar plates are inoculated with a standardized inoculum of the test microorganism. Then, some wells (about 5 mm in diameter) are made into the solid medium to place the samples and the controls to test at a desired concentration. The Petri dishes are incubated under suitable conditions. Generally, the antimicrobial agent diffuses into the agar and inhibits germination and growth of the test microorganism. The diameters of inhibition growth zones are measured.

- What are the conclusions of this work?

Answer: Based on the obtained results, it was concluded that it was possible to synthetize Zn-ACF with overall good yields. Moreover, it was possible to confirm the Zn-ACF formation by FTIR and PXRD. Additionally, the antimicrobial activity of the obtained Zn-ACF was evaluated. Results showed higher antimicrobial activity of Zn-ACF than the starting material, nalidixic acid, suggesting that small changes in a chemical structure can result in great biological differences.

Instructor notes for the experiments

- At the end of this step, the students are expected to understand how to do and read an antimicrobial activity study.
- For logistic reasons, we suggest the preparation of the microbial suspensions in advance.

Preparation of microbial suspensions

1. In Petri dishes containing 20 mL of medium (Mueller-Hinton agar), inoculate the respective microorganisms with a sterilized loop.
2. After 24h of incubation at 37°C for microorganisms to grow, prepare the inoculum suspension of *E. coli* (ATCC 25922).
3. In a sterile bidistilled water tube, prepare each microbial suspension using a swab and adjust it to obtain a final concentration of 0.5 McFarland standard. (Note: McFarland standard is used as a reference to adjust the turbidity of bacterial suspensions). $BaSO_4$ turbidity is equivalent to a McFarland 0.5 standard solution: Add an aliquot of 0.5 mL of $BaCl_2$ 0.048 mol / L (1.175% (w) / v $BaCl_2 \cdot 2H_2O$) to 99.5 mL of H_2SO_4 0.18 mol / L (1% v /v) stirring constantly.
4. The previous microbial suspensions are ready to perform the well diffusion test (note: Petri dishes with Mueller-Hinton agar are also needed to perform each antimicrobial evaluation).

References

1. André, V.; Quaresma, S.; da Silva, J. L. F.; Duarte, M. T. Exploring mechanochemistry to turn organic bio-relevant molecules into metal-organic frameworks: a short review. *Beilstein J Org Chem* **2017**, *13*, 2416-2427.

2. PubChem Zinc nitrate hexahydrate. https://pubchem.ncbi.nlm.nih.gov/compound/15865313.

3. PubChem Nalidixic Acid. https://pubchem.ncbi.nlm.nih.gov/compound/4421.

4. PubChem Ammonia solution 25% (v/v). https://pubchem.ncbi.nlm.nih.gov/compound/222#section=GHS-Classification.

5. PubChem Ethanol. https://pubchem.ncbi.nlm.nih.gov/compound/702#section=Safety-and-Hazards.

6. Aldrich, S. GHS EU Poster . https://www.sigmaaldrich.com/content/dam/sigma-aldrich/docs/promo_NOT_INDEXED/General_Information/1/h_overview.pdf.

7. Aldrich, S. Overview Precautionary Statements. . https://www.sigmaaldrich.com/content/dam/sigma-aldrich/docs/promo_NOT_INDEXED/General_Information/1/p_overview.pdf.

8. Griffiths, P. R.; De Haseth, J. A. Chapter 1: Introduction to Vibrational Spectroscopy. In *Fourier Transform Infrared Spectrometry* **2006**; pp 1-18.

9. Law, D.; Zhou, D. Chapter 3 - Solid-State Characterization and Techniques. In *Developing Solid Oral Dosage Forms*Elsevier Inc: **2017**; pp 59-84.

10. Escola de Ciências e Tecnologias da Saúde-Universidade Lusófona de Humanidades e Tecnologías *Medicinal Inorganic Chemistry Manual Practical Lessons;* Universidade Lusófona de Humanidades e Tecnologías: Lisbon, Portugal, 2020-2021.

11. Clinical and Laboratory Standards Institute *M100-S25 Performance Standards for Antimicrobial Susceptibility Testing: Twenty-Fifth Informational Supplement;* Clinical and Laboratory Standards Institute: Wayne, Pennsylvania, USA, **2015**, pp 238.

12. Magaldi, S.; Mata-Essayag, S.; Hartung de Capriles, C.; Perez, C.; Colella, M. T.; Olaizola, C.; Ontiveros, Y. Well diffusion for antifungal susceptibility testing. *Int J Infect Dis* **2004**, *8 (1)*, 39-45.

13. O'Connell, K. M. G.; Hodgkinson, J. T.; Sore, H. F.; Welch, M.; Salmond, G. P. C.; Spring, D. R. Combating Multidrug-Resistant Bacteria: Current Strategies for the Discovery of Novel Antibacterials. *Angew Chem Int Ed Engl* **2013**, *52 (41)*, 10706-10733.

14. Pham, T. D. M.; Ziora, Z. M.; Blaskovich, M. A. T. Quinolone antibiotics. *MedChemComm* **2019**, *1*, 1719-1739.

15. Balouiri, M.; Sadiki, M.; Ibnsouda, S. K. Methods for *in vitro* evaluating antimicrobial activity : A review. *J Pharm Anal.* **2016**, *6*, 71-79.

16. PubChem Dimethylsulfoxide. https://pubchem.ncbi.nlm.nih.gov/compound/Dimethyl-sulfoxide.

17. ATCC *Escherichia coli*. https://webcache.googleusercontent.com/search?q=cache:NHWulAd1hLEJ:https://www.atcc.org/~/ps/25922.ashx+&cd=3&hl=es&ct=clnk&gl=es.

18. University of Illinoisis Required Training for work at Biosafety Level 1. https://drs.illinois.edu/Page/Programs/RegistrationInformation#RequiredTraining.
